# Ossicular Chain Reconstruction With Glass Ionomer Cement Following Removal of Active Middle Ear Implant

**DOI:** 10.1002/oto2.70062

**Published:** 2025-01-03

**Authors:** William J. McFeely, Alexis E. McFeely, Jack A. Shohet

**Affiliations:** ^1^ North Alabama ENT Associates P.C. Huntsville Alabama USA; ^2^ UAB Heersink School of Medicine Birmingham Alabama USA; ^3^ Shohet Ear Associates Medical Group Inc. Orange County California USA

**Keywords:** active middle ear implant, audiometry, Esteem®, explant, glass ionomer cement, ossiculoplasty, rebridging, reconstruction

## Abstract

The use of bone cement in ossicular chain reconstruction (OCR) represents an area of recent interest. This multi‐institutional retrospective study assesses the efficacy of glass ionomer cement (GIC) in OCR following the explantation of a fully implantable active middle ear implant. A postoperative 4‐frequency mean air‐bone gap (ABG) was obtained for 15 subjects by averaging 0.5, 1, 2, and 4 kHz frequencies. For Group A (short‐term, N = 15), at a mean of 4.5 months postoperatively, 9 (60%) achieved an ABG between 0 and 10 dB, 5 (33%) were 11 to 20 dB, and 1 (7%) was 21 to 30 dB. For Group B (long‐term, N = 5), at a mean of 50 months postoperatively, 4 (80%) were 0 to 10 dB and 1 (20%) was 11 to 20 dB. These results suggest that GIC represents an effective means of ABG closure after device explantation.

Though commonly recognized as an effective dental restorative material, glass ionomer cement (GIC) has emerged as a powerful tool in ossicular chain reconstruction (OCR). The use of this acid‐base cement in incudostapedial ossiculoplasty (ISO) has been reported in the restoration of conductive hearing loss resulting from chronic ear diseases, including chronic otitis media (COM).^1^ The literature highlights the use of GIC in incudostapedial rebridging ossiculoplasty in cases of erosion due to chronic ear disease to reduce the air‐bone gap (ABG).^2^ Bone cement is comparable to conventional rebridging techniques (ie, autografts and prostheses), with most subjects achieving postoperative ABG ≤ 20 dB.^3^, ^4^

Despite research to support the use of GIC in OCR for patients with COM, there are no reports demonstrating the use of this material exclusively for ISO following explantation of a fully implantable active middle ear implant (AMEI) (Esteem®; Envoy Medical Corporation). Analysis of the postoperative ABG of patients undergoing AMEI removal followed by ISO using GIC provides the unique opportunity to assess this technique in a controlled setting (closure of a consistent distal lenticular process defect).

## Methods

**Figure 1:**
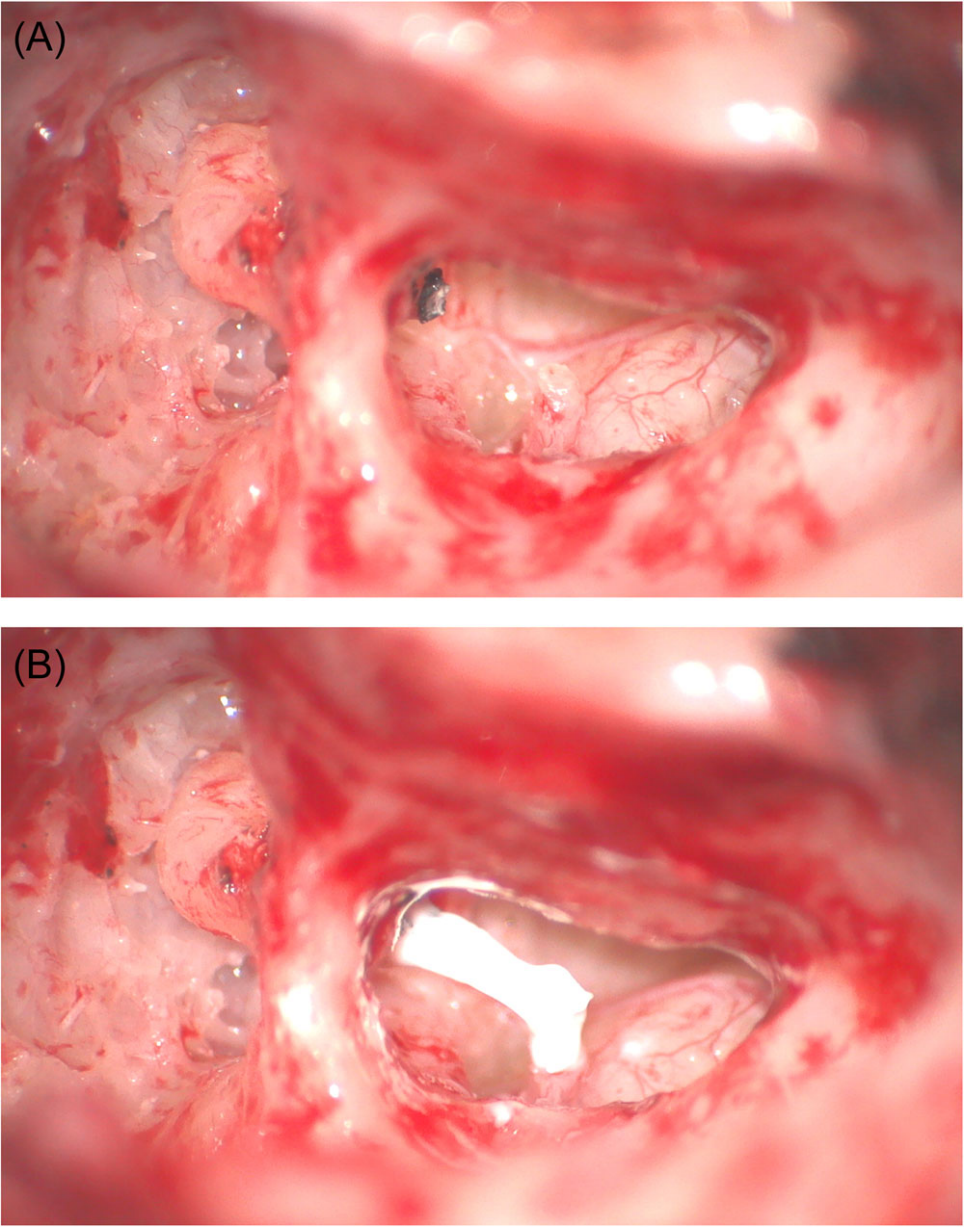
Intraoperative photographs demonstrating (A) incudostapedial discontinuity after AMEI explantation and before rebridging, and (B) reconstructed ossicular chain following ISO with GIC. AMEI, active middle ear implant; GIC, glass ionomer cement; ISO, incudostapedial ossiculoplasty.

**Figure 2:**
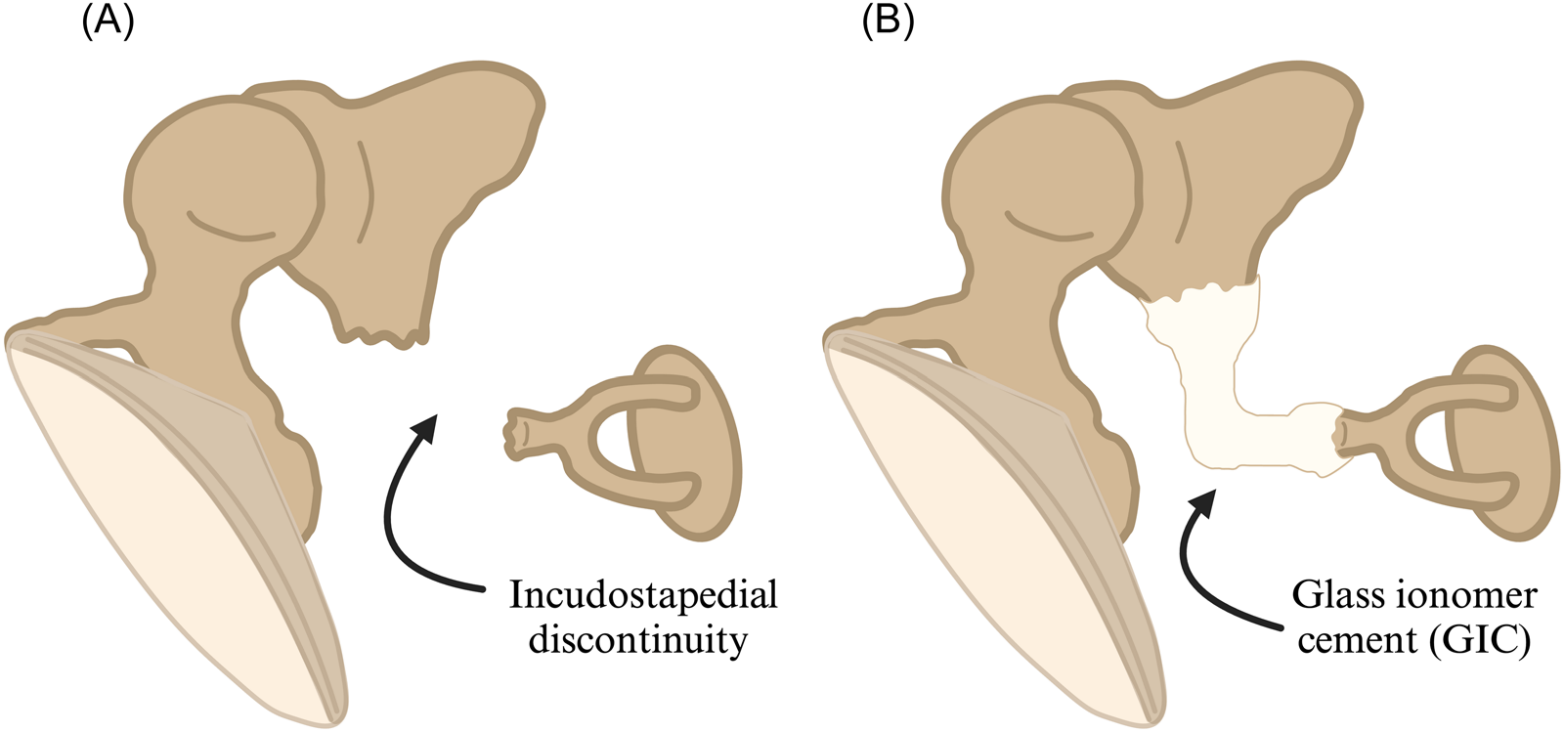
Illustrations demonstrating (A) incudostapedial discontinuity after AMEI explantation and before rebridging, and (B) reconstructed ossicular chain following ISO with GIC. Created with BioRender.com. AMEI, active middle ear implant; GIC, glass ionomer cement; ISO, incudostapedial ossiculoplasty.

Fifteen subjects (N = 15) who had undergone an ISO using EnvoyCem GIC (Envoy Medical Corporation) to reconstruct a 3 to 4 mm surgically induced distal lenticular process defect following explantation of an Esteem® AMEI between June 2013 and June 2023 in either Huntsville, Alabama or Orange County, California were identified for inclusion in this study. Each subject underwent removal of the AMEI using an extended transmastoid facial recess approach (Figures 1A and 2A) followed by ISO with GIC (Figures 1B and 2B).

Postoperative audiometric data were reviewed to calculate individual ABG at 0.5, 1, 2, and 4 kHz as well as a 4‐frequency mean ABG. Subjects were categorized into 2 groups based on the timing of the postoperative audiogram. Group A (short‐term) included all 15 subjects, each having a postoperative audiogram between 3 and 12 months. Group B (long‐term) represented a subset (N = 5) of Group A whose most recent audiogram was at least 18 months after surgery. Adverse events were reviewed. Microsoft Excel software was employed for statistical analysis.

Subjects who failed to return for audiometric testing between 3 and 12 months postoperatively, and those with other middle ear pathology at surgery were excluded.

Institutional Review Board (IRB) exemption was obtained through WCG IRB on July 26, 2024.

## Results

**Figure 3:**
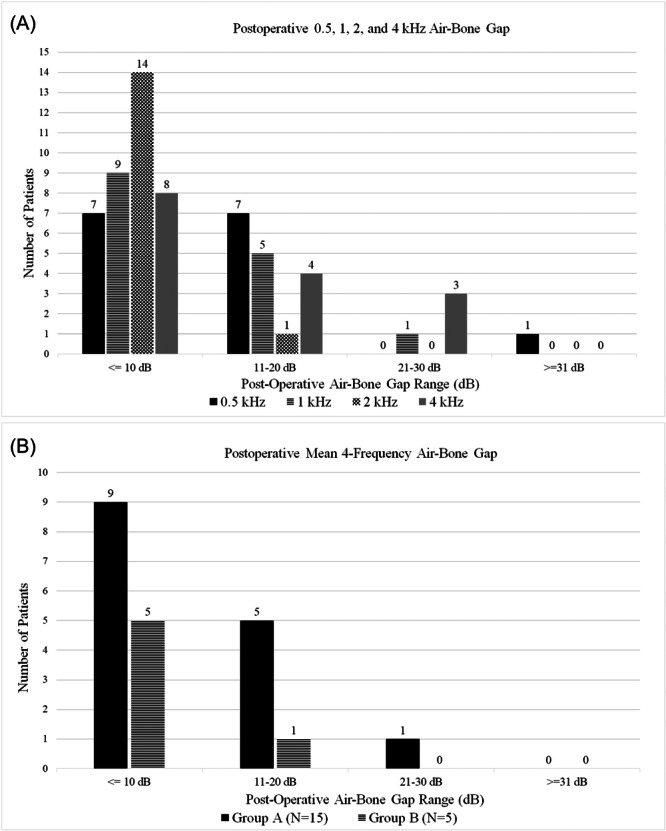
Graphical depiction of (A) Group A postoperative ABG at 0.5, 1, 2, and 4 kHz separated into 0 to 10, 11 to 20, 21 to 30, and ≥31 dB ranges, and (B) categorization of mean 4‐frequency ABG for Group A and B subjects into 0 to 10, 11 to 20, 21 to 30, and ≥31 dB. ABG, air‐bone gap.

At the time of ISO, the average age of Group A subjects was 63 years old (range 25‐83). There were 5 female and 10 male patients; surgery was performed on 10 left and 5 right ears. The first postoperative audiogram for Group A subjects was obtained at an average of 4.5 months (range 3‐12 months, SD 2.3) postoperatively. Of the 15 patients, 9 (60%) achieved a mean 4‐frequency ABG of 0 to 10 dB, 5 (33%) were 11 to 20 dB, and 1 (7%) was 21 to 30 dB (Figure 3B). Individual averages for each frequency were 13 dB (range 0‐45, SD 11.1) for 500 Hz, 11.3 dB (range 0‐25, SD 6.5) for 1 kHz, 6 dB (range 0‐20, SD 5.8) for 2 kHz, and 14.3 dB (range 0‐30, SD 7.9) for 4 kHz. The overall mean 4‐frequency ABG for all subjects in Group A was 11.2 dB (range 5‐27.5, SD 5.2) (Table 1).

**Table 1:** Air‐Bone Gap Data for Group A (All Subjects) and Group B at 0.5, 1, 2, and 4 kHz

Patient number	Group ID	ABG at 0.5 kHz, dB	ABG at 1 kHz, dB	ABG at 2 kHz, dB	ABG at 4 kHz, dB
1	A	0	10	0	10
2	A	15	10	0	15
3	A	20	15	10	5
4	A	15	0	5	10
5	A	5	10	20	20
6	A	10	15	0	15
7	A	5	10	10	0
8	A	15	10	10	20
9	A	15	10	0	10
10	A	20	5	10	25
11	A	10	0	5	10
12	A	20	15	0	10
13	A	0	15	0	25
14	A	45	25	10	30
15	A	0	20	10	10
1	B	10	5	10	15
2	B	5	5	10	15
3	B	10	0	5	20
4	B	20	15	10	15
5	B	5	10	5	5

Group B included 1 female and 4 male subjects at an average of 69 years old (range 58‐78). At an average of 50 months after surgery (range 19‐64 months, SD 15.8), 4 (80%) had a mean 4‐frequency ABG of 0 to 10 dB and 1 (20%) was between 11 and 20 dB (Figure 3B). Individual averages for each frequency were 10 dB (range 5‐20, SD 5.5) for 500 Hz, 7 dB (range 0‐15, SD 5.1) for 1 kHz, 8 dB (range 5‐10, SD 2.5) for 2 kHz, and 14 dB (range 5‐20, SD 4.9) for 4 kHz. The overall mean 4‐frequency ABG for all subjects included in Group B was 9.8 dB (range 6‐15, SD 2.9) (Table 1).

The *χ* ^2^ test was used to evaluate the difference between groups when comparing those reaching an effective ABG (defined as ≤20 dB). The proportion of subjects with an effective postoperative ABG ≤ 20 dB did not differ between Groups A and B, *χ* ^2^ (1, N = 20) = 0.35, *P* = .55.

There were no perioperative complications, although 1 subject (7%) did require revision OCR due to incomplete fusion of the GIC with the incus 7 months later.

## Discussion

Several techniques exist to achieve incudostapedial rebridging and restore conductive hearing for isolated lenticular process incus defects. Among them, GIC represents a safe and effective option for ossiculoplasty during chronic ear surgery.^1^, ^2^, ^3^, ^4^, ^5^ In fact, bone cement may be more effective in producing a lower postoperative ABG compared with prostheses and autografts.^3^

A distal incus defect typically results from chronic ear diseases such as COM or cholesteatoma, and its repair often includes concomitant tympanoplasty. In this study, we had the unique opportunity to repair this isolated ossicular defect without tympanoplasty or the presence of chronic disease. This study is the first to describe the exclusive use of GIC for ISO in this setting.

The results support the use of GIC for ISO upon AMEI removal, further highlighting the versatility of this substance. Nearly all patients from Groups A and B (N = 14/15 and N = 5/5 respectively) achieved an effective postoperative ABG ≤ 20 dB; it is reasonable to conclude that GIC is an effective means of OCR. These results compare favorably with the findings of other bone cement OCR systematic review studies.^3^, ^4^ Our findings are similar to a hydroxyapatite study which demonstrated ABG ≤ 20 dB in 83% of subjects at least 6 months after surgery.^6^ Our overall mean 4‐frequency ABG for all subjects in Group A was 11.2 dB and Group B was 9.8 dB. These results are comparable to those found using the Kraus K‐Helix Crown prosthesis with GIC in chronic ears in which the mean postoperative ABG was 10.5 dB.^7^

Long‐term hearing results remained stable in this study, showing no statistically significant change between groups A and B. The mean individual frequency ABG for Group A was lowest (6 dB) at 2 kHz compared with the other frequencies studied. This finding is relevant, as sounds centered around 2 kHz are critical for speech perception, clarity, and intelligibility.^8^

The retrospective nature of this case series and noncontrolled design represent study limitations. Both groups were small in size, limiting the power of statistical analysis. This report does support the need for a larger more powerful study, and our results are consistent with other GIC studies (not associated with AMEI removal) concerning ABG. Expanding study inclusion criteria to subjects with (1) a distal lenticular defect from chronic ear disease (not just after AMEI explantation), and (2) utilizing surgical approaches such as transcanal, would allow for a more powerful analysis of this specific GIC technique for ISO.

## Conclusions

This study indicates that ISO with GIC is an effective means of ABG closure at the time of AMEI explantation. Both the short‐term and long‐term data support the use of this substance for incudostapedial joint reconstruction and compare favorably with hydroxyapatite alone as well as the K‐Helix Crown prosthesis. This report is the first to describe a series of patients who underwent ISO only using GIC without tympanoplasty via the transmastoid facial recess approach. This research further highlights the reversibility of a specific implantable AMEI by demonstrating a reliable method of ISO to restore hearing conduction.

## Author Contributions

**William J. McFeely Jr**, study design, data acquisition, analysis and interpretation of data, revision of the manuscript, performed surgery on 11 subjects; **Alexis E. McFeely**, writing of the manuscript, presentation of the research, figure design; **Jack A. Shohet**, study design, data acquisition, revision of the manuscript for important intellectual content, performed surgery on 4 subjects.

## Disclosures

### Competing interests

William J. McFeely Jr discloses that he is a consultant for Stryker Instruments, a division of Stryker Corporation. Jack A. Shohet discloses that he is a member of the Envoy Medical Advisory Board. Alexis E. McFeely declares that she has no conflict of interest.

### Funding source

None.
